# Sympathetic neuropathology is revealed in muscles affected by amyotrophic lateral sclerosis

**DOI:** 10.3389/fphys.2023.1165811

**Published:** 2023-05-12

**Authors:** Antonio Mazzaro, Veronica Vita, Marco Ronfini, Irene Casola, Arianna Klein, Gabriella Dobrowolny, Gianni Sorarù, Antonio Musarò, Marco Mongillo, Tania Zaglia

**Affiliations:** ^1^ Department of Cardiac, Thoracic, Vascular Sciences and Public Health, University of Padua, Padua, Italy; ^2^ Veneto Institute of Molecular Medicine, Padua, Italy; ^3^ Department of Biomedical Sciences, University of Padua, Padua, Italy; ^4^ Laboratory Affiliated to Institute Pasteur Italia-Fondazione Cenci Bolognetti, DAHFMO-Unit of Histology and Medical Embryology, Sapienza University of Rome, Rome, Italy; ^5^ Department of Neuroscience, Azienda Ospedaliera di Padova, Padua, Italy; ^6^ Scuola Superiore di Studi Avanzati Sapienza (SSAS), Sapienza University of Rome, Rome, Italy; ^7^ CNR Institute of Neuroscience, Padua, Italy; ^8^ CIR-MYO Myology Center, University of Padua, Padua, Italy

**Keywords:** amyotrophic lateral sclerosis, sympathetic neurons, skeletal muscle innervation, SOD1^G93A^ mutation, sympathetic neurodegeneration

## Abstract

**Rationale:** The anatomical substrate of skeletal muscle autonomic innervation has remained underappreciated since it was described many decades ago. As such, the structural and functional features of muscle sympathetic innervation are largely undetermined in both physiology and pathology, mainly due to methodological limitations in the histopathological analysis of small neuronal fibers in tissue samples. Amyotrophic lateral sclerosis (ALS) is a fatal neuromuscular disease which mainly targets motor neurons, and despite autonomic symptoms occurring in a significant fraction of patients, peripheral sympathetic neurons (SNs) are generally considered unaffected and, as such, poorly studied.

**Purpose:** In this research, we compared sympathetic innervation of normal and ALS muscles, through structural analysis of the sympathetic network in human and murine tissue samples.

**Methods and Results:** We first refined tissue processing to circumvent methodological limitations interfering with the detection of muscle sympathetic innervation. The optimized “Neuro Detection Protocol” (NDP) was validated in human muscle biopsies, demonstrating that SNs innervate, at high density, both blood vessels and skeletal myofibers, independent of the fiber metabolic type. Subsequently, NDP was exploited to analyze sympathetic innervation in muscles of SOD1^G93A^ mice, a preclinical ALS model. Our data show that ALS murine muscles display SN denervation, which has already initiated at the early disease stage and worsened during aging. SN degeneration was also observed in muscles of MLC/SOD1^G93A^ mice, with muscle specific expression of the SOD1^G93A^ mutant gene. Notably, similar alterations in SNs were observed in muscle biopsies from an ALS patient, carrying the SOD1^G93A^ mutation.

**Conclusion:** We set up a protocol for the analysis of murine and, more importantly, human muscle sympathetic innervation. Our results indicate that SNs are additional cell types compromised in ALS and suggest that dysfunctional SOD1^G93A^ muscles affect their sympathetic innervation.

## 1 Introduction

Amyotrophic lateral sclerosis (ALS) is a fatal neuromuscular disorder, hallmarked by motor neuron (MN) degeneration, with progressive weakness of voluntary muscles and respiratory failure, the latter representing the main cause of death, which typically occurs within 4–5 years from diagnosis. The highly heterogeneous presentation of ALS is not entirely predictable by the disease cause, which may be sporadic (s) or familial (f) (Couratier et al., 2016; Grollemund et al., 2019; Tarlarini et al., 2019). ALS-linked mutations, responsible for fALS forms, have been identified in more than 30 genes, including *SOD1* (which accounts for up to 12%–23% of fALS) (Bosco et al., 2010), *TDP-43* (Sreedharan et al., 2008), *FUS* (Vance et al., 2009), and *C9ORF72* (Smith et al., 2013). At the time being, the pathogenesis of ALS is elusive and an mechanistic insight is strikingly poor, resulting in the absence of mechanism-driven therapies capable of preventing or attenuating the dramatic disease progression (Karch et al., 2009; Milanese et al., 2014; Tripolszki et al., 2017; Mejzini et al., 2019).

For several decades after its early description, ALS has been regarded as the result of a pathologic process confined to MNs. Recently, however, evidence of non-neuronal cell types directly participating in disease development upheld the definition of a “multicellular” disorder. The concept, mainly emerging from the studies on monogenic fALS, is forwarded upon appreciation that while disease mutations can lead to toxic gain-of-function in MNs, the neuronal fate (i.e., degeneration and death) is influenced by the expression of mutations in cells which interact, with various biological roles, with MNs, including astrocytes (Clement et al., 2003; Yamanaka et al., 2008; Ziff et al., 2022). Remarkably, while transgenic mice ubiquitously expressing fALS-associated SOD1 mutants (e.g., G93A, G37R, and G85R) develop a rapidly progressive disease, with lower MN degeneration, reminiscent of human ALS (Gurney et al., 1994; Dal Canto and Gurney, 1995; Wong et al., 1995; Mourelatos et al., 1996; Bruijn et al., 1997), selective expression of the same mutations in MNs did not cause significant cell death. Along the same lines, modulation of the cytotoxic effects of the SOD1 mutation in glial cells was suggested to prevent MN death in the context of ALS (Pramatarova et al., 2001; Ambrożkiewicz et al., 2022; Perez-Gonzalez et al., 2022). The converging evidence suggests that the progression of the pathologic phenotype in fALS requires coordinated expression of the disease mutation in both MNs and non-neuronal cells, including oligodendrocytes (Kang et al., 2013), astrocytes (Clement et al., 2003; Haidet-Phillips et al., 2011), and microglia (Milanese et al., 2014; Quek et al., 2022), advanced the hypothesis that mutation-dependent neurotoxicity originates, with diverse mechanisms, by alterations in the cellular environment surrounding MNs. Interestingly, recent findings of the Musarò Laboratory, exploiting cell-specific targeting of the SOD1 ALS mutation G93A, demonstrated that the aberrant activation of redox cascades in skeletal myocytes underlies an additional mechanism, leading to morphological alterations in the neuromuscular presynaptic terminals and neuromuscular junction (NMJ) dismantlement (Dobrowolny et al., 2018). To further increase the complexity of the ALS picture, SOD1^G93A^ mice have shown defects in cell-to-cell adhesion between the endothelial cells of the blood–spinal cord barrier, and a recent study supports a central contribution of the SOD1-mutant-mediated endothelial damage to disease initiation that may accumulate prior to MN degeneration and neurovascular inflammatory response (Zhong et al., 2008). Such an already wide spectrum of cells participating in the disease may further extend upon appreciation that subclinical alterations in autonomic control of organ functions are frequently present in ALS patients and that the early manifestations of ALS occur in peripheral tissues and involve muscle atrophy and weakness, NMJ alterations, and metabolic changes (Shimizu et al., 1994; Baltadzhieva et al., 2005; Pavlovic et al., 2010; Merico and Cavinato, 2011; Shimizu et al., 2011; Shimizu, 2013; Dalla Vecchia et al., 2015; De Maria et al., 2015; Piccione et al., 2015; Nolano et al., 2017; Rosenbohm et al., 2017; Pimentel et al., 2019; Silveira et al., 2022). Analysis of heart rate variability (HRV) (Pimentel et al., 2019; Brown et al., 2022) and systolic arterial pressure variability (SAPV) revealed altered sympatho-vagal balance (Moreau et al., 2012; Xia et al., 2022), and microneurography demonstrated a reduced spontaneous activity of sympathetic efferences in sciatic nerves, suggestive of neurodegeneration (Donadio and Liguori, 2015). The same trial has demonstrated that SOD1^G93A^ mice show a 24% decrease in TH expression in adrenal glands (Kandinov et al., 2013). Although it is long known that sympathetic axons, running within motor nerves, project to skeletal muscles and interact with both myocytes and vasculature (Boeke, 1909a; Boeke, 1909b; Boeke, 1913; Barker and Saito, 1981; Chan-Palay et al., 1982; Tadaki et al., 1995; Grasby et al., 1997), and the architecture and function of such non-myelinated adrenergic fibers are commonly disregarded. Only recently, high-resolution immunofluorescence imaging and *in vivo* second messenger microscopy of nerves and muscles have shown that catecholamine-releasing sympathetic varicosities, which contact the myocyte sarcolemma in proximity to the NMJ (Khan et al., 2016; Straka et al., 2018), activate myocyte β-adrenergic receptors (β-ARs), influencing muscle performance, intracellular protein turnover, and NMJ homeostasis (Rudolf et al., 2014; Khan et al., 2016; Rodrigues A. C. Z. et al., 2019; Rodrigues A. Z. C. et al., 2019; Rodrigues et al., 2021a; Rodrigues et al., 2021b; Delbono et al., 2021; Wang et al., 2022). Remarkably, we found that retrograde neurotrophic signaling from target cells to sympathetic neurons (SNs) is required to maintain autonomic innervation of highly innervated organs like the heart (Dobrowolny et al., 2018; Dokshokova et al., 2022), surmising that the interruption of bidirectional trophic communication between sympathetic nerves and muscles may negatively synergize on innervation.

Collectively, evidence suggests that autonomic neurons, which were considered uninjured in the classic disease description, may indeed be additional targets in ALS, but this has remained largely unexplored.

On these bases, in this study, we aimed to characterize the state of sympathetic innervation of different skeletal muscles and determine whether the arrangement and cellular morphology of SNs are altered in ALS. To reach this goal, we refined an immunofluorescence method to reveal sympathetic nerves, which we applied to muscle tissue samples from wild-type and SOD1^G93A^ ALS mice, at different disease stages. Results were compared with those obtained from muscles of mice carrying the SOD1^G93A^ mutation selectively in the skeletal muscle (MLC/SOD1^G93A^ mice) and controlled with the analysis of sympathetic innervation in muscle biopsies from control healthy subjects and ALS patients.

## 2 Methods

### 2.1 Ethical approval

Experimental procedures in murine models have been approved by the Ministry of Health (Ufficio VI), in compliance with the Animal Welfare Legislation (protocols A06E0.N.ERD and A06E0.18). All procedures were performed by trained personnel with documented formal training and previous experience in experimental animal handling and care. All procedures were refined prior to initiating the study, and the number of animals was calculated to use the least number of animals sufficient to achieve statistical significance, according to sample power calculations.

### 2.2 Origin and source of animals

We used 2- and 5-month-old B6SJ-Tg(SOD1^G93A^)1Gur/J transgenic male mice (Charles River), 5-month-old MLC/SOD1^G93A^ male mice (Dobrowolny et al., 2018), and age- and sex-matched littermate controls. Animals were maintained in individually ventilated cages in an authorized animal facility (authorization numbers 609/2015-PR and 864/2020-PR) under a 12:12 h light/dark cycle at a controlled temperature and allowed access to water and food *ad libitum.*

### 2.3 Muscle fixation and immunofluorescence analysis on thin murine muscle sections

Mice were sacrificed by cervical dislocation, and the hind-leg muscles were carefully excised, fixed in 1% paraformaldehyde (PFA) for 30 min at room temperature (RT), dehydrated in a sucrose gradient, and frozen in liquid nitrogen. A cryostat (Leica CM 1850, Leica Microsystems GmbH, Wetzlar, Germany) was used to obtain 10-μm muscle sections, and the sections were processed for immunofluorescence, following the protocol described by Straka et al. (2018). Primary antibodies used in this study are listed in Supplementary Table S1.

### 2.4 Human muscle biopsy processing and immunofluorescence analysis

Muscle biopsies were collected after obtaining written informed consent, using an open biopsy procedure. For ALS patients, biopsies were taken from either the biceps or vastus lateralis muscles, while for control subjects, biopsies were taken from vastus lateralis. A subset of biopsies was fixed in 1% PFA for 30 min at RT, dehydrated in a sucrose gradient, and frozen in liquid nitrogen. A cryostat (Leica CM 1850, Leica Microsystems GmbH, Wetzlar, Germany) was used to obtain 10-μm muscle sections, and the sections were processed for immunofluorescence, following the protocol described by Straka et al. (2018). Primary antibodies used in this study are listed in Supplementary Table S1.

### 2.5 Whole-mount immunofluorescence of murine muscle fibers and confocal microscopy analysis

A subset of murine muscles was processed with whole-mount immunofluorescence. In detail, muscles were briefly fixed in 4% PFA. Fiber bundles were dissected, incubated in 50 mM NH_4_Cl for 30 min at RT, and permeabilized with 0.5% Triton X-100 for 4 h at RT. Fibers were incubated with anti-tyrosine hydroxylase (TH) overnight at 4°C. Once washed in phosphate-buffered saline (PBS), fibers were incubated with the appropriate secondary antibody for 2 h at RT (Straka et al., 2018) and subsequently with Alexa Fluor 488-conjugated α-bungarotoxin (1:200, Invitrogen) for 2 h at RT. Fibers were analyzed using the confocal microscope (Leica TCS SP5, Leica Microsystems, Germany), equipped with LAS AF software, using a ×63/1.25 oil objective. Z-stacks were generated from images taken at 0.4-μm intervals, and maximum intensity projections and 3D reconstructions were obtained using ImageJ software (version 1.53q, National Institutes of Health, Bethesda, MD, United States).

### 2.6 Morphometric analyses

In murine muscles, the sympathetic neuron density was calculated in six non-consecutive longitudinal sections from both murine and human muscles. For each section, eight randomly chosen images were acquired and analyzed using ImageJ software (version 1.53q, National Institutes of Health, Bethesda, MD, United States). Neuronal density was expressed as the percentage of a muscle area occupied by TH-positive fibers.

### 2.7 Statistical analysis

Statistical analysis was performed using GraphPad Prism 9. The normality of data distribution was assessed with the Shapiro–Wilk test. The unpaired *t*-test (for two groups) or one-way ANOVA (for three or more groups), with Kruskal–Wallis and Dunnett’s tests, was used for normally distributed data. The unpaired *t*-test with Welch’s correction was used to compare two groups with normally distributed data and unequal variance. A *p*-value < 0.05 was considered statistically significant.

## 3 Results

### 3.1 Optimization of tissue processing for sympathetic neuron detection in murine and human skeletal muscles

**FIGURE 1:**
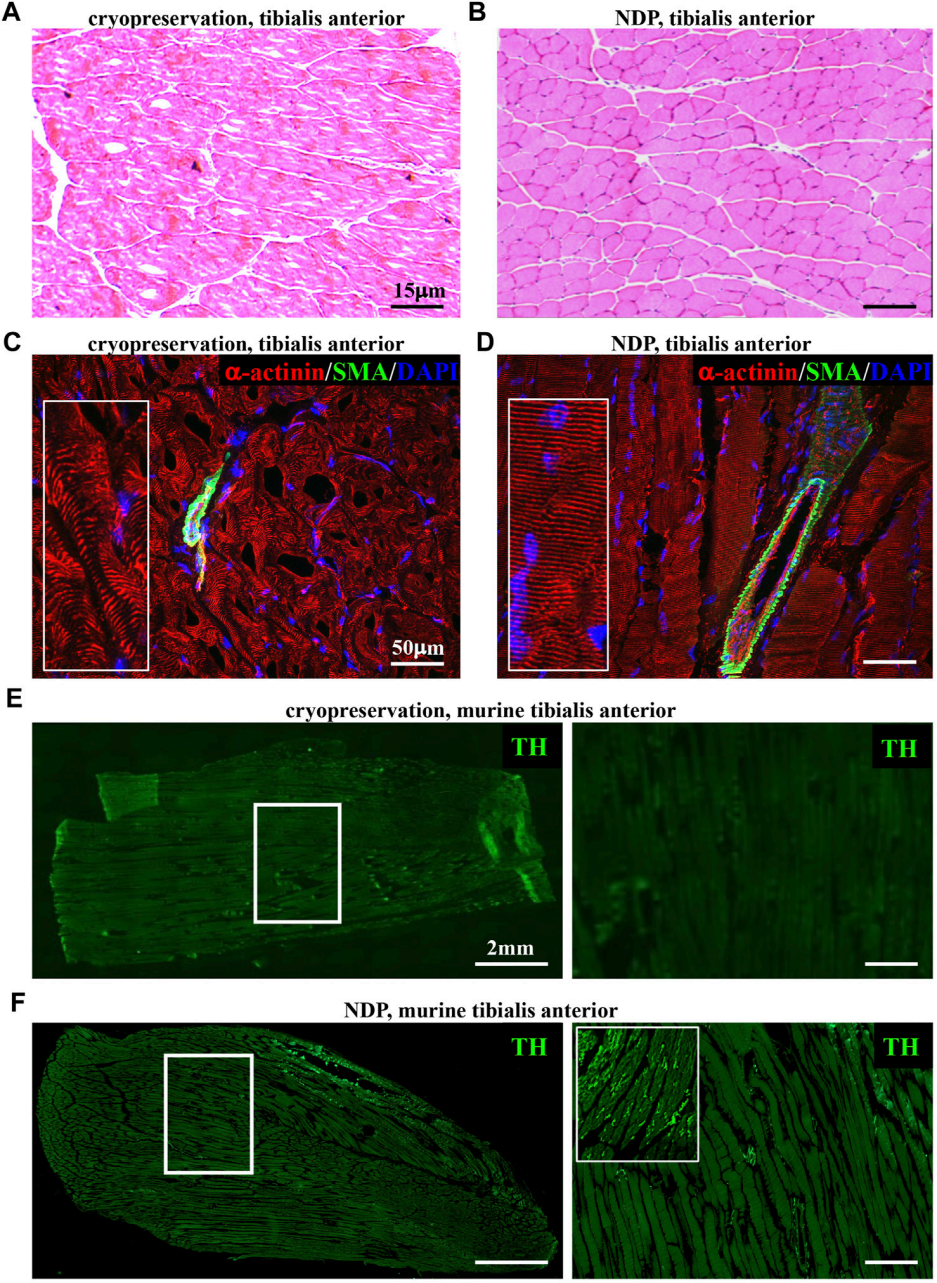
Histopathology of normal murine muscles processed with Neuron Detection Protocol (NDP). **(A, B)** Hematoxylin–eosin staining of mouse tibialis anterior sections that underwent standard cryopreservation **(A)** or NDP **(B)**. **(C, D)** Immunofluorescence analysis of cryopreserved **(C)** vs*.* NDP-processed **(D)** tibialis anterior sections, co-stained with antibodies to sarcomeric α-actinin (red) and smooth muscle actin (SMA, green). Nuclei were counterstained with DAPI (blue). **(E, F)** Immunofluorescence analysis of cryopreserved **(E)** vs*.* NDP-processed **(F)** tibialis anterior sections, stained with an antibody to tyrosine hydroxylase (TH, green). The right panels show high magnifications of the white boxes in the left images. We analyzed 16 muscles (n = 8 cryopreserved and n = 8 processed with NDP) harvested from eight adult C57BL6/J male mice.

The identification of structural neuropathological alterations in skeletal muscles has frequently been overlooked for the difficulties in preserving the small and fragile neuronal processes in commonly used standard histological preparations (i.e., muscle biopsies). Our experience with the assessment of cardiac sympathetic innervation in thin sections prompted us to refine tissue processing protocols, previously used in heart and skeletal muscles (Zaglia et al., 2013; Straka et al., 2018), to inspect the topology of SN processes in murine hind-limb muscles (i.e., tibialis anterior, soleus, gastrocnemius, and quadriceps), using confocal immunofluorescence. To this aim, muscles were harvested from adult C57BL6/J mice and fixed with different PFA concentrations (1, 2 e 4%, v:v in PBS) and fixation time (from 10 min to 2 h, at room temperature), dehydrated in a sucrose gradient, and frozen in liquid nitrogen. Longitudinal thin cryosections were subsequently analyzed upon hematoxylin–eosin staining and confocal immunofluorescence with sarcomere and neuronal markers, including small fiber-specific markers (i.e., tyrosine hydroxylase), revealing that 1% PFA fixation for 30 min is the most suited combination to allow optimal tissue preservation and fluorescence microscopy imaging. Such processing protocols [which we here referred to as Neuron Detection Protocol (NDP), for readability] allow preservation of tissue morphology and architecture, including the interactions among myocytes, vascular bed, neurons, and interstitium (Figures 1A, B; Supplementary Figure S1). Figure 1 shows that by comparing panels **C** (cryopreserved section) and **D** (fixed section), α-actinin-stained sarcomeres are more regularly distributed along the myocyte length and resolved with the lower background signal and higher image contrast in fixed sections. In addition, while neurofilament-positive neurons are detectable with both processing protocols, small-sized and fragile structures, such as TH-positive SN processes, were intact and almost exclusively detectable in fixed muscle sections (Figures 1E, F; Supplementary Figure S2). As further usefulness, aggressive tissue unmasking is not required (i.e., microwave unmasking) for immunofluorescence, and the protocol overcomes the need to adjust PFA concentration and fixation time for different muscle types, as the chosen parameters were applicable to both small- (i.e., soleus) and large-sized (i.e., gastrocnemius and quadriceps) murine muscles.

**FIGURE 2:**
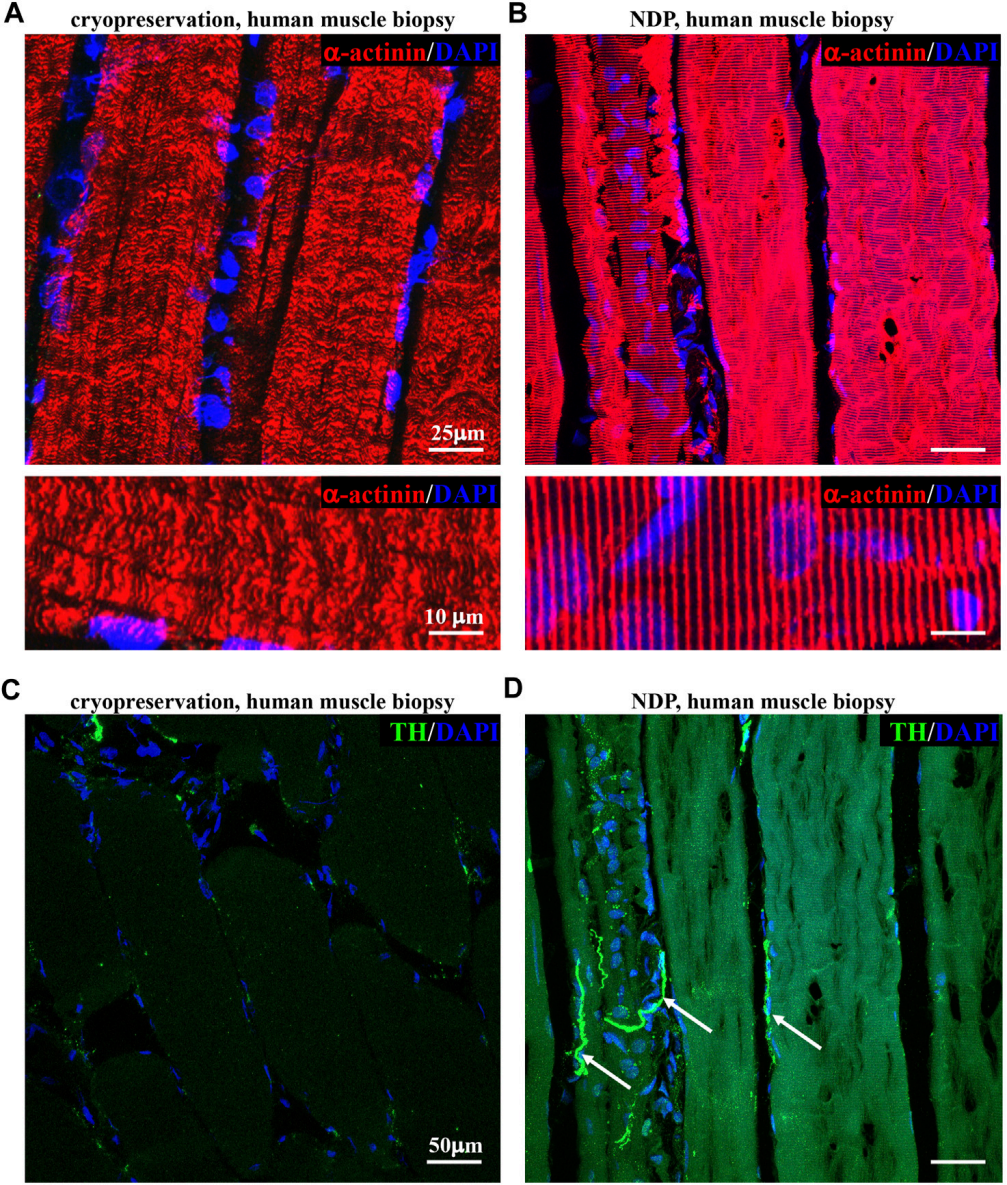
Histopathology of healthy human muscles processed with Neuron Detection Protocol . **(A, B)** Confocal immunofluorescence analysis of cryopreserved **(A)** vs*.* NDP-processed **(B)** sections of vastus lateralis muscle biopsies from healthy subjects, stained with an antibody to sarcomeric α-actinin (red). Nuclei were counterstained with DAPI (blue). **(C, D)** Confocal immunofluorescence analysis of cryopreserved **(C)** vs*.* NDP-processed **(D)** sections of vastus lateralis muscle biopsies from healthy subjects, stained with an antibody to tyrosine hydroxylase (TH, green). Nuclei were counterstained with DAPI (blue). Arrows in **(D)** indicate sympathetic neurons (SNs). We analyzed muscle biopsies from five healthy controls.

We thus tested whether such variants of fixation protocol are suitable for processing and histological analysis of human muscle biopsies, a procedure widely used in the diagnostic workup of neuromuscular diseases, including ALS. To this aim, we compared tissue morphology and immunofluorescence staining in 1 mm^3^ biopsies from healthy volunteers, which underwent either direct freezing or tissue processing as described previously for murine muscles (see *Methods* section). Remarkably, muscles prepared with our optimized protocol appeared structurally intact and better preserved than the conventionally processed counterparts. Figure 2 shows that the application of “NDP” allowed more detailed immunofluorescence imaging of human muscle cytoarchitecture, which is well suited for the histopathological study of sarcomeres and innervation, including that mediated by small fibers, such as SNs. Notably, the latter appears to be almost absent in biopsies that underwent standard processing.

Thus, NDP represents a useful strategy for comprehensive morphological analysis of muscle samples and the assessment of both myocyte and non-myocyte components, including the poorly studied sympathetic nerves, in both preclinical models and human biopsies.

### 3.2 Sympathetic innervation of different murine hind-limb muscles

**FIGURE 3:**
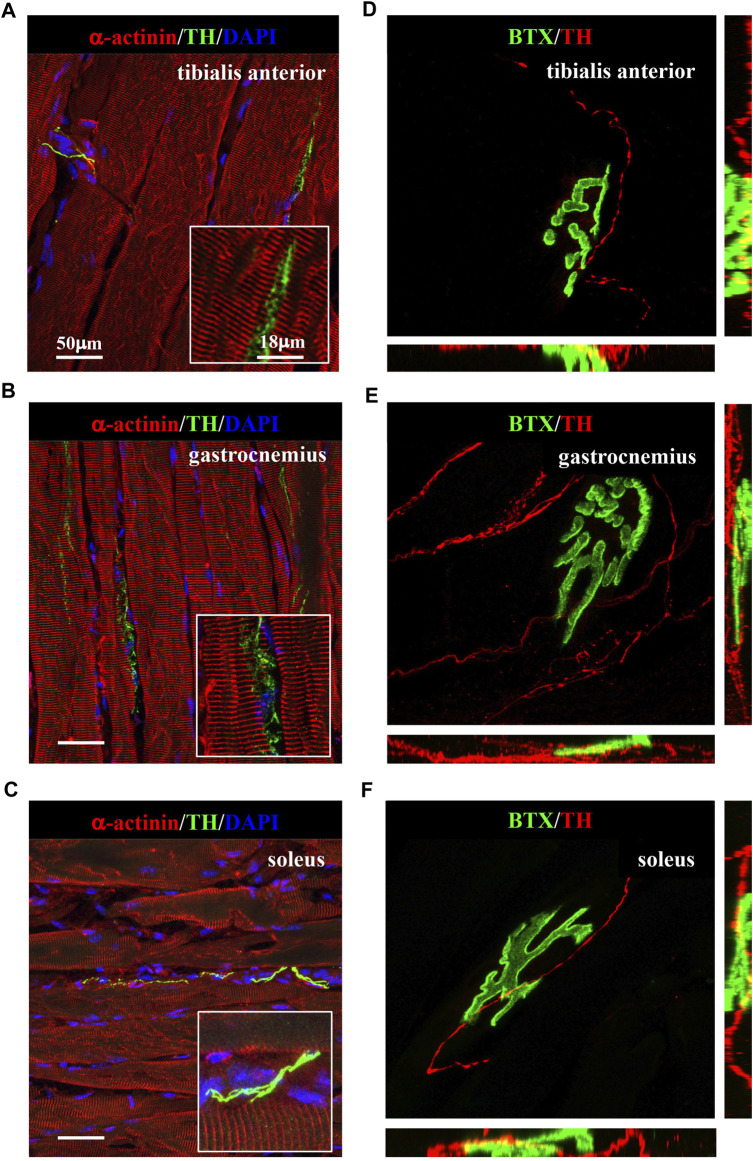
Sympathetic innervation topology in normal murine muscles. **(A–C)** Confocal immunofluorescence in muscle sections of tibialis anterior **(A)**, gastrocnemius **(B)**, and soleus **(C)**, processed with NDP, co-stained with antibodies to tyrosine hydroxylase (TH, green) and sarcomeric α-actinin (red). Nuclei were counterstained with DAPI (blue). **(D–F)** Three-dimensional rendering of confocal optical sections of isolated muscle fibers from tibialis anterior **(D)**, gastrocnemius **(E)**, and soleus **(F)** that underwent whole-mount immunofluorescence with Alexa Fluor 488-conjugated bungarotoxin (BTX, green) and an antibody to tyrosine hydroxylase (TH, red). We analyzed muscles harvested from eight adult C57BL/6J male mice.

**FIGURE 4:**
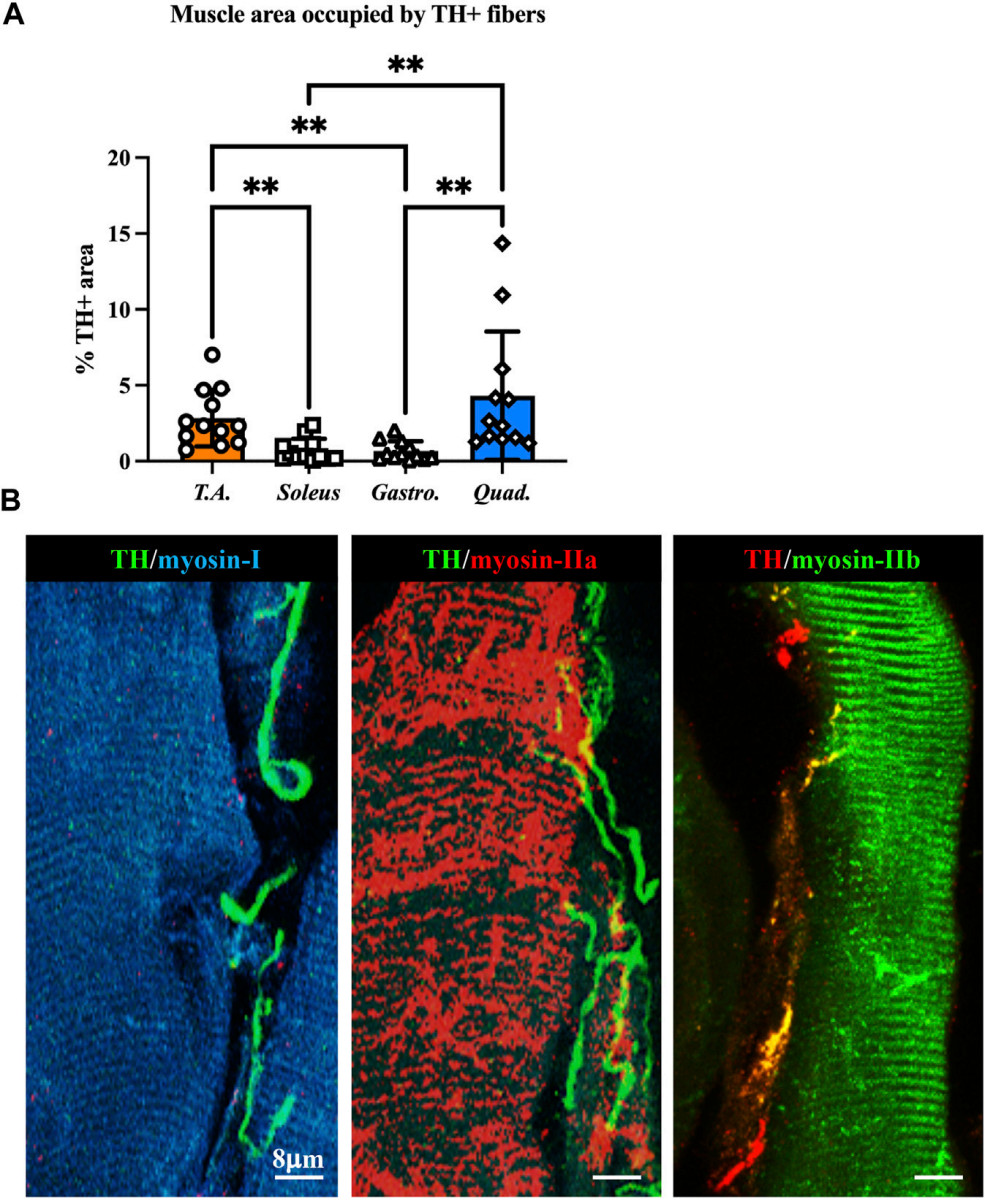
Characterization of sympathetic innervation in different muscle types from normal mice. **(A)** Quantification of sympathetic innervation density in different muscles, evaluated as the fractional area occupied by TH-positive fibers. Bars indicate s.d. Differences among groups were determined using Kruskal–Wallis and Dunnett’s tests for multiple comparisons (**, *p* < 0.01. Each value plotted in the graph represents the average neuronal density calculated from different images acquired from one individual muscle, as detailed in the Methods section. For each muscle type, we analyzed 12 samples, harvested from six different adult C57BL6/J male mice). **(B)** Confocal immunofluorescence in NDP-processed tibialis anterior sections, co-stained with antibodies to tyrosine hydroxylase (TH) in combination with anti-myosin-I (left panel), anti-myosin-IIa (middle panel), and anti-myosin-IIb (right panel).

We thus applied the muscle processing protocol, described previously, to analyze the microanatomy of sympathetic innervation in different muscles (i.e., tibialis anterior, soleus, gastrocnemius, and quadriceps) of wild-type mice. Confocal co-immunofluorescence using anti-TH in combination with antibodies to either smooth muscle actin or sarcomeric α-actinin was used to label vascular smooth muscle cells and muscle sarcomeres, respectively. In line with the previous research (Khan et al., 2016; Straka et al., 2018), SN processes were found in all muscle types analyzed, both around blood vessels and in close apposition with skeletal myocytes, although the interaction between the sections of SN processes and these two target structures appeared to be different (Figures 3A–C; Supplementary Figure S3). Indeed, while vessel-interacting SN processes embrace the vascular wall, the muscle-interacting SN processes accompany the myocyte fiber for most of its length and, as previously described by Straka et al. (2018), are in close contact with the sarcolemma in correspondence to the NMJ (Figures 3D–F). After delineating the SN topology, we assessed the density of sympathetic innervation in different muscles, with morphometric analysis of immunofluorescence images. The fractional area of the muscle section occupied by SNs varied among the different muscles analyzed and was the highest in the quadriceps and lower in soleus and gastrocnemius (Figure 4A). To determine whether such differences could be attributed to the different muscle fiber types, anti-TH-stained sections were labeled with isoform-specific antibodies for myosin, to discriminate slow vs*.* IIa and IIb fast fibers. As shown in Figure 4B, muscle SNs establish a similar interaction regardless of the fiber metabolic type.

**FIGURE 5:**
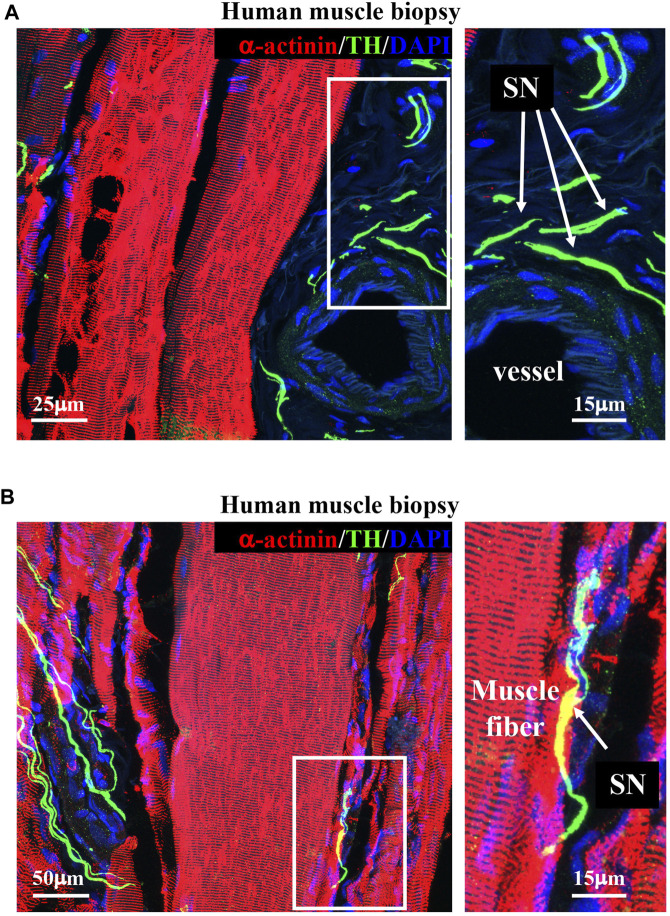
Sympathetic innervation topology in healthy human muscles. **(A, B)** Confocal immunofluorescence in sections of human vastus lateralis biopsies, processed with NDP and co-stained with antibodies to tyrosine hydroxylase (TH, green) and sarcomeric α-actinin (red). Nuclei were counterstained with DAPI (blue). Right images show high magnifications of the white boxes in the left panels. Arrows in **(A, B)** indicate sympathetic neurons. We analyzed muscle biopsies from five healthy controls.

Upon determining the morphology of muscle sympathetic efferences in mice, we sought to verify whether human muscles displayed similar features. To this aim, we performed confocal immunofluorescence on muscle biopsies collected from healthy volunteers, which confirmed the general aspects of sympathetic innervation topology, with some neuronal processes sprouted around blood vessels and others in close apposition with muscle fibers (Figure 5).

### 3.3 Sympathetic neuropathology in muscles from ALS (SOD1^G93A^) mice

**FIGURE 6:**
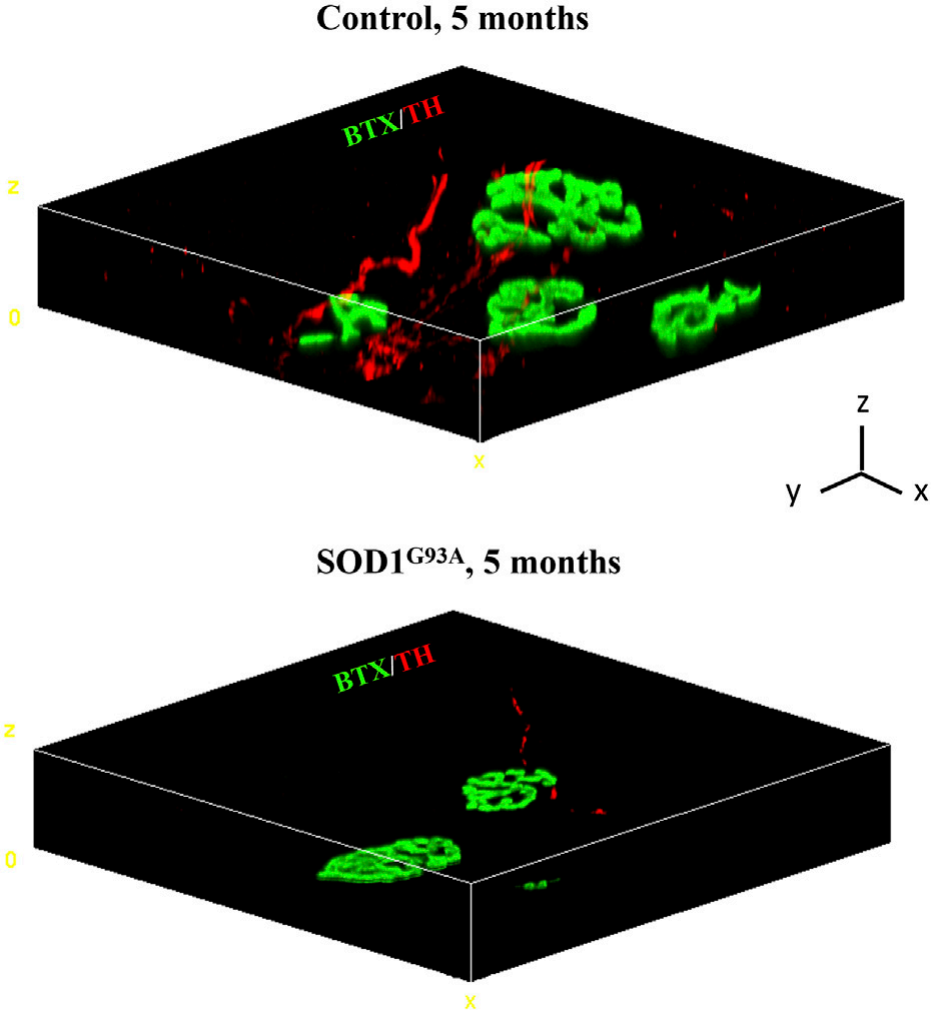
Three-dimensional reconstruction of the sympathetic neuron network in normal vs*.* SOD1^G93A^ muscle fibers. Three-dimensional-rendering of SNs and NMJ in confocal optical sections of whole muscle fibers isolated from the tibialis anterior of a 5-month-old control (top panel) or SOD1^G93A^ (bottom panel) mice, processed as described by Straka et al. (2018), and whole-mount stained with Alexa Fluor 488-conjugated bungarotoxin (BTX, green) and an antibody to tyrosine hydroxylase (TH, red). Images are representative of the analysis of n = 6 control muscles and n = 6 SOD1^G93A^ muscles, harvested from n = 6 control mice and n = 6 ALS mice, respectively. Scale bars (x, y, and z) correspond to 15 μm.

A growing number of reports suggests that catecholaminergic signaling is required for the maintenance of muscle health, and impaired SN/skeletal myocyte communication has been described in several neuromuscular disorders (Rudolf et al., 2013; Rudolf et al., 2014; Khan et al., 2016; Rodrigues A. C. Z. et al., 2019; Rodrigues A. Z. C. et al., 2019; Rodrigues et al., 2021a; Rodrigues et al., 2021b; Delbono et al., 2021). The observation that SNs are in structural and functional connection with MNs and skeletal muscles prompted us to test whether SNs could be abnormal in ALS, a fatal neurodegenerative disease associated with MN degeneration, muscle atrophy, and paralysis, with unexplained signs of subclinical dysautonomia (Shimizu et al., 1994; Baltadzhieva et al., 2005; Pavlovic et al., 2010; Merico and Cavinato, 2011; Shimizu et al., 2011; Shimizu, 2013; Dalla Vecchia et al., 2015; De Maria et al., 2015; Piccione et al., 2015; Nolano et al., 2017; Rosenbohm et al., 2017; Pimentel et al., 2019; Silveira et al., 2022). To ascertain our hypothesis, we assessed the state of sympathetic innervation in skeletal muscles from SOD1^G93A^ mice, a well-accepted preclinical model of ALS (Gurney et al., 1994). First, we processed isolated muscle fibers (i.e., tibialis anterior, soleus, quadriceps, and gastrocnemius) from control and SOD1^G93A^ ALS transgenic mice that were harvested at an advanced disease stage (5 months) and characterized by MN degeneration and muscle paralysis (Chiu et al., 1995; Fischer et al., 2004). Three-dimensional imaging revealed that, in ALS muscles, SNs interacting with the NMJ, which were as expected altered in size and density (Dupuis and Loeffler, 2009), were almost undetectable, with only very few thin and fragmented TH-positive processes (Figure 6).

**FIGURE 7:**
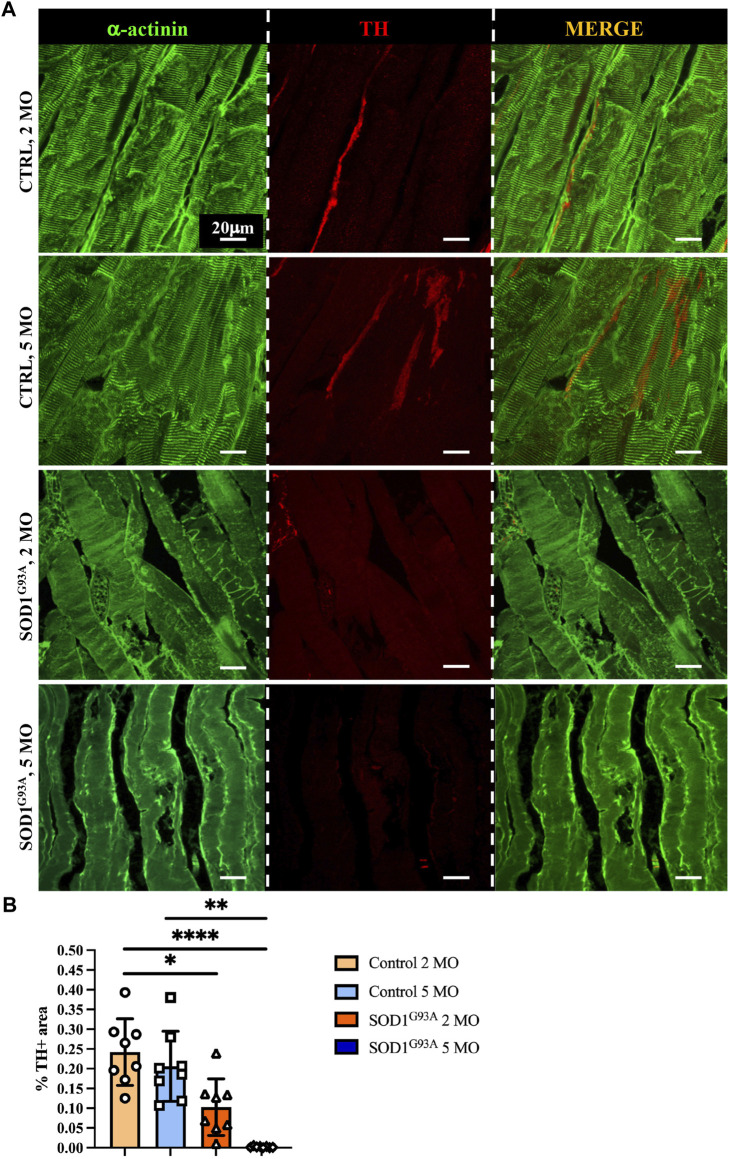
Sympathetic neuropathology in SOD1^G93A^ ALS muscles. **(A)** Confocal immunofluorescence of quadriceps sections from 2- and 5-month-old control and SOD1^G93A^ mice, NDP processed and co-stained with antibodies to sarcomeric α-actinin (left panels, green) and tyrosine hydroxylase (TH, middle panels, red). The right panels show the merged images. **(B)** Quantification of sympathetic innervation density in quadriceps from control and ALS mice, at different disease stages. SN density was evaluated as the fractional area occupied by TH-positive fibers. Bars indicate s.d. Differences among groups were determined using a one-way ANOVA with Dunnett’s test for multiple comparisons (*, *p* < 0.05; **, *p* < 0.01; ****, *p* < 0.0001; each value plotted in the graph represents the average neuronal density calculated from different images acquired from one individual muscle, as detailed in the Methods section. For each group, we analyzed eight muscles harvested from four different mice).

To define whether SN alterations are selectively evident at the advanced disease stage or SNs are already altered at the stage before an overt disease and to relate SN degeneration with disease progression, we thus compared SN density of thin sections of muscles from SOD1^G93A^ mice, at 2 and 5 months of age, corresponding to early and advanced stages of disease (Chiu et al., 1995; Fischer et al., 2004). We here provided a comprehensive morphometric evaluation of muscle sympathetic innervation in sections, co-stained with anti-TH and anti-sarcomeric actinin, of quadriceps, which, based on our previous analyses (see Figure 5), has the highest sympathetic innervation density. Our results show that significant sympathetic denervation occurs in SOD1^G93A^ quadriceps, with a global decrease in SN innervation density at an early timepoint and a complete denervation in the overt disease phase (Figures 7A, B). Similar results were obtained in different muscles, including soleus, tibialis anterior, and gastrocnemius, identifying sympathetic neuropathology as an additional disease process in ALS.

### 3.4 Sympathetic neuropathology in muscles from mice with muscle-restricted SOD1^G93A^ expression

**FIGURE 8:**
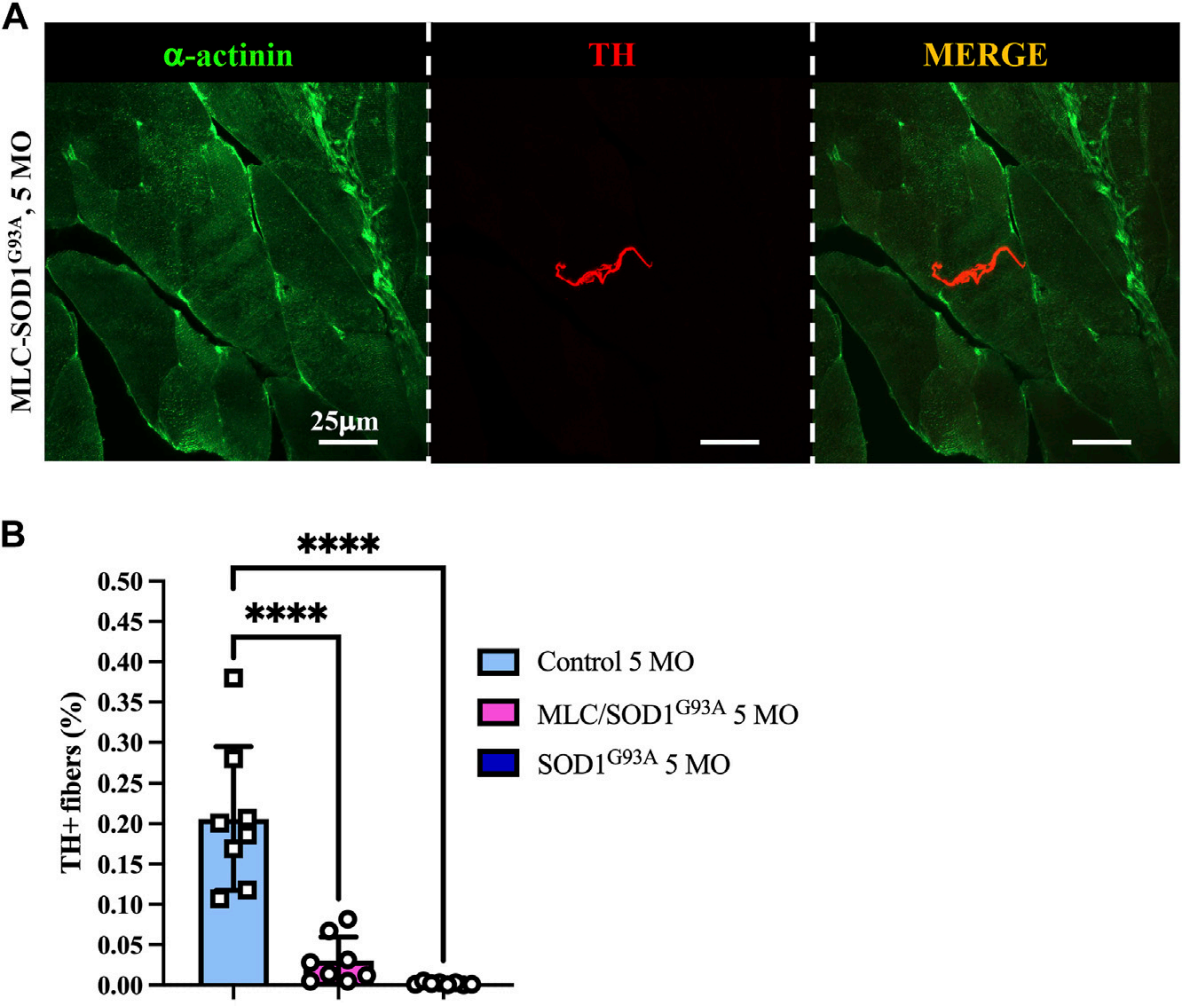
Sympathetic neuropathology in MLC/SOD1^G93A^ muscles. **(A)** Confocal immunofluorescence of quadriceps sections, processed with NDP, from 5-month-old MLC/SOD1^G93A^ mice. Sections were co-stained with antibodies to sarcomeric α-actinin (left panels, green) and tyrosine hydroxylase (TH, middle panels, red). The right panels show the merged images. **(B)** Quantification of sympathetic innervation density in quadriceps from 5-month-old control, SOD1^G93A^, and MLC/SOD1^G93A^ mice. SN density was evaluated as the fractional area occupied by TH-positive fibers. Bars indicate s.d. Differences among groups were determined using a one-way ANOVA with Dunnett’s test for multiple comparisons (*, *p* < 0.05; **, *p* < 0.01; ****, *p* < 0.0001; each value plotted in the graph represents the average neuronal density calculated from the different images acquired from one individual muscle, as detailed in the Methods section. For each group, we analyzed eight muscles harvested from four different mice).

Muscle sympathetic degeneration in SOD1^G93A^ mice may be attributed to 1) the direct neurotoxic effect of SOD1^G93A^ mutation on SNs, 2) an indirect effect of ALS muscles on innervating neurons, or 3) a combination of both events. Based on our previous demonstration that muscle-restricted expression of SOD1^G93A^ leads to alterations at the spinal cord level and reminisces some of the disease phenotype, we compared the state of sympathetic innervation in muscle-specific MLC/SOD1^G93A^ vs*.* ubiquitous-SOD1^G93A^ mice. Muscles were analyzed at 5 months of age, a stage characterized, in MLC/SOD1^G93A^ mice, by muscle atrophy, NMJ dismantlement, metabolic alteration, and microglia activation (Dobrowolny et al., 2018). Interestingly, confocal immunofluorescence imaging and morphometric analysis showed that MLC/SOD1^G93A^ muscles have altered SN process morphology and decreased sympathetic innervation density, which were qualitatively and quantitatively comparable to those observed in muscles from SOD1^G93A^ mice (Figure 8).

### 3.5 Sympathetic innervation is altered in human SOD1^G93A^ ALS muscle

**FIGURE 9:**
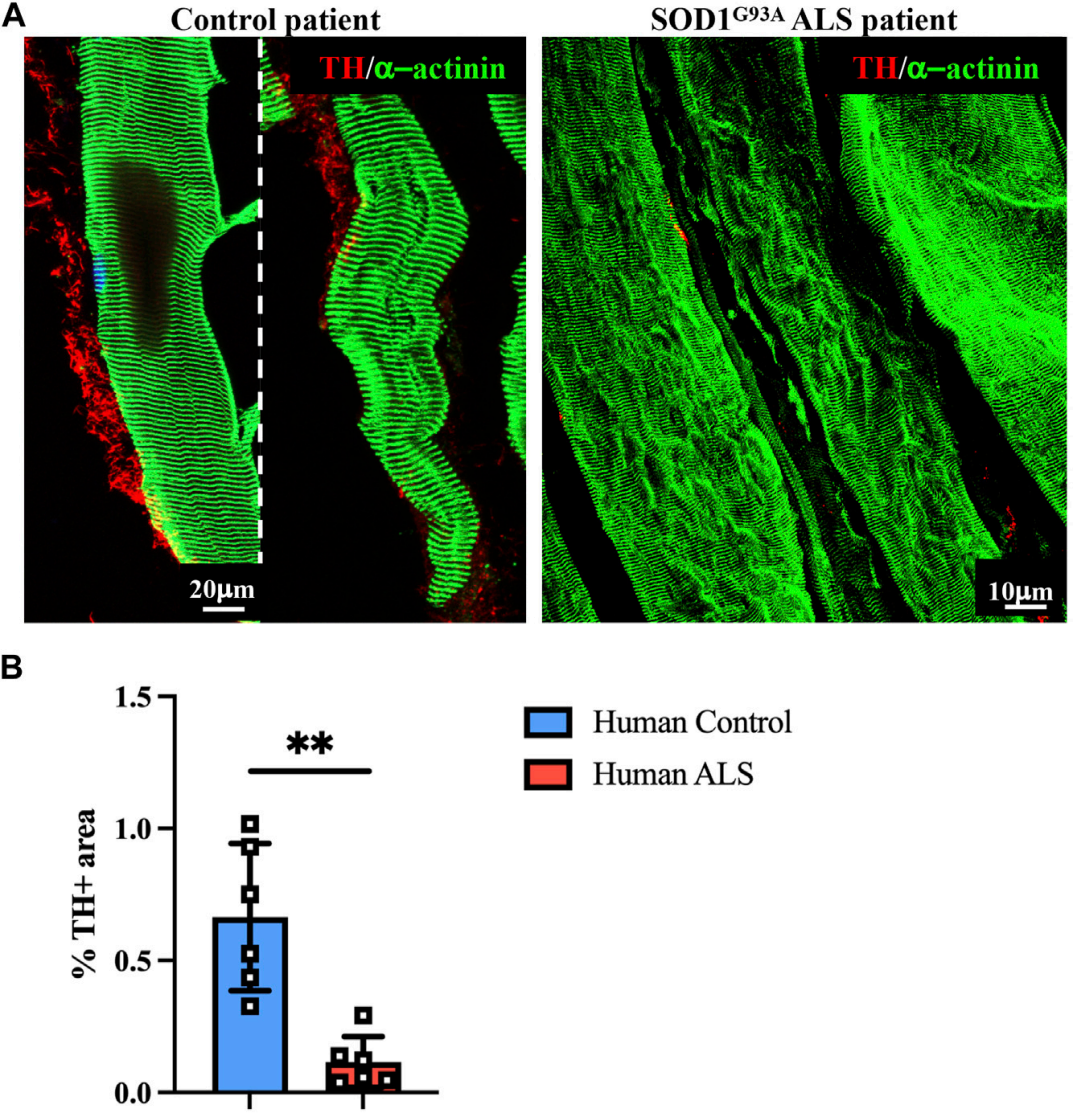
Sympathetic neuropathology in human SOD1^G93A^ muscle. **(A)** Confocal immunofluorescence in sections of NDP-processed vastus lateralis muscle biopsies from a control subject (male, 55 years, HyperCPKemia) and a fALS patient (male, 58 years, SOD1^G93A^ mutation). Sections were co-stained with antibodies to tyrosine hydroxylase (TH, red) and sarcomeric α-actinin (green). **(B)** Quantification of sympathetic innervation density in muscle biopsies from control and ALS subjects in **(A)**. SN density was evaluated as the fractional area occupied by TH-positive fibers. Bars indicate s.d. Differences among groups were determined using an unpaired *t*-test (**, *p* < 0.01; values plotted in the graph represent the average neuronal density in n = 6 non-consecutive sections of muscle biopsy).

To evaluate whether the impaired muscle sympathetic innervation, observed in ALS mice, is also recognizable in patients, we performed a proof-of-concept analysis on muscle biopsies from a control healthy subject vs*.* a patient diagnosed with ALS caused by SOD1^G93A^ mutation, with chronic neurogenic atrophy and myopathic signs. Muscle biopsies were processed with NDP, and thin sections were stained with sarcomeric α-actinin- and TH-specific antibodies. Notably, confocal immunofluorescence evidenced, in ALS muscle, a dramatic alteration in the morphology of SN processes, which appeared fragmented with reduced axonal sprouting, all features strikingly similar to those observed in SOD1^G93A^ and MLC/SOD1^G93A^ murine muscles (Figure 9A). Moreover, innervation density appeared to be significantly reduced, and these data were confirmed by the quantification of the muscle area occupied by SN processes (Figure 9B), which was roughly to the same degree as what was observed in the preclinical ALS models.

## 4 Discussion

ALS is primarily associated with the degeneration of MNs, which plays a critical role in the development of the typical symptoms of the disease (Gurney et al., 1994; Tarlarini et al., 2019; Corcia et al., 2021). Although MN death is believed to result from a combination of rather unspecific mechanisms, including protein accumulation, mitochondrial dysfunction, and oxidative stress, the disease shows remarkable cell specificity, as the number of primary neuronal targets is relatively small (Karch et al., 2009; Milanese et al., 2014; Mejzini et al., 2019). Peripheral autonomic neurons, including sympathetic and parasympathetic types, form a largely represented population in most body tissues (Scalco et al., 2021) but are generally considered unaltered in ALS, despite clinical evidence accruing that autonomic symptoms can be revealed in a significant fraction of patients. Interestingly, SNs interact with skeletal myofibers and have recently been shown to impinge on both neuromuscular transmission and myocyte proteostasis (Khan et al., 2016; Straka et al., 2021; Wang et al., 2022), both of which are key processes in ALS-associated neuromuscular failure and myopathy (Khan et al., 2016; Dobrowolny et al., 2018). This prompted us to address whether SNs may be additional neuronal types affected in ALS. We first aimed to circumvent methodological limitations which may interfere with the detection of muscle sympathetic innervation, by refining the tissue processing procedures to allow optimal preservation of the complex tissue architecture and cytological features of the diverse cell types forming the muscle, including the small and fragile SNs. The protocol was thus validated in muscle tissues harvested from mouse and human muscle biopsies and subsequently applied to define the topology and density of sympathetic innervation and neuro-muscular interactions in different hind-limb muscle fragments. Furthermore, we exploited the protocol to analyze sympathetic innervation in muscles from SOD1^G93A^ mice, a commonly studied preclinical ALS model, and from fALS patients, collectively showing that muscle SN processes appear degenerated, when already at early disease stages, and may thus be regarded as an additional cell type affected in the disease.

The autonomic nervous system, which is formed by the sympathetic and parasympathetic branches, controls over organism homeostasis by continuously delivering regulatory inputs to almost all the innervated tissues. In the case of neuro-muscular interactions, autonomic neurons, and in particular SNs, have long been shown to innervate both the working myocardium and skeletal muscles (Straka et al., 2018; Pianca et al., 2019; Di Bona et al., 2020). In the heart, the effects of neurogenic regulation are undoubted and manifest with the perceptible increase in rate and contraction force, during physiological (e.g., emotions and exercise) or pathological (e.g., pressure overload and hypovolemia) stresses (Zaglia and Mongillo, 2017; Franzoso et al., 2022). Despite the effects of sympathetic activation on the cardiovascular system reflecting on important clinical consequences (i.e., syncope and arrhythmias) and yielding several methods to infer neuronal function (e.g., blood pressure, heart rate variability, cardiovascular effect of postural changes, scintigraphy, and PET), the characteristics of myocardial sympathetic innervation were only recently appreciated upon investigation of cardiac neurons with enhanced tools and refined techniques (for reviews on the topic, see Di Bona et al., 2020; Scalco et al., 2021). Recent findings have included the appreciation of the unexpected density of heart innervation (Pianca et al., 2019), the mechanisms of hetero-cellular neuro-cardiac communication (Prando et al., 2018; Dokshokova et al., 2022), and additional constitutive roles of SNs in the modulation of cell division, proteostasis, and electrophysiology (Ogawa et al., 1992; Kanevskij et al., 2002; O’Connell et al., 2003; Zaglia et al., 2013; Kreipke and Birren, 2015; Pianca et al., 2019).

The evidence that SNs are present in skeletal muscles dates over a century ago (Boeke, 1909a; Boeke, 1909b; Boeke, 1913), but the main targets of muscle-innervating neurons remained, for a long time, confined to blood vessels. Although innervation of intra- and extra-fusal fibers of several muscles was observed in the early 1980s (Barker and Saito, 1981), the lack of evident effects of SNs on myocyte function and pathology and the disagreement on experimental findings have somewhat detracted from progress in sympatho-muscular research. Only recently, muscle SNs were reappraised, and the use of improved methodologies (e.g., imaging and transgenic mice) undoubtedly demonstrated that, in addition to vascular cells, a still undetermined fraction of SN processes also targets skeletal myofibers and is poised to control a broad range of muscle functions beyond blood perfusion, including neuro-muscular synaptic transmission, proteostasis, and intracellular protein trafficking (Khan et al., 2016; Straka et al., 2018; 2021; Rodrigues A. C. Z. et al., 2019; Rodrigues A. Z. C. et al., 2019; Rodrigues et al., 2021a; Rodrigues et al., 2021b; Delbono et al., 2021; Wang et al., 2022). In addition, the evidence of the biochemical and structural effects of sympathectomy, as well as those of pharmacological modulation of the molecular targets of SNs in myocytes (i.e., β-adrenergic receptors) (Navegantes et al., 2002; Rudolf et al., 2013; Wang et al., 2022) further supports the existence of a population of SNs interacting with muscle cells.

Despite the evidence of such diverse roles of muscle SNs in physiology, and the numerous indications of their involvement in myopathies and neuro-muscular disorders, progress in understanding muscle autonomic control has been discontinuous (Edgeworth, 1930; Schara et al., 2009; Lashley et al., 2010; Liewluck et al., 2011; Meinen et al., 2012; Finlayson et al., 2013; Rudolf et al., 2013; Khan et al., 2016; Clausen et al., 2018; McMacken et al., 2018; 2019; Webster et al., 2020; Delbono et al., 2021). Such disregard may have been influenced by the methodological problems which hindered the transparent detection of SNs in muscles. In contrast with the heart, where SNs dominate the neuronal population of the myocardial landscape, in muscles, the stage is taken by larger-sized MNs, which underlay voluntary control of contraction (Mendell, 2005). However, we and others observed that preservation of the thin and fragile SNs within the tissue, for analytical purposes, requires dedicated processing protocols in both skeletal and cardiac muscles, and as such, commonly used histological routines may lead to underappreciation of the SN population. In the first place, physical–chemical factors are associated with the tissue, in which preparation may affect SN cytoarchitecture. SN processes are made of small-sized unmyelinated fibers (Zaglia and Mongillo, 2017; Franzoso et al., 2022), and the absence of a lipid layer is expected to increase vulnerability to the effects of freezing, e.g., ice crystal formation, which would only minimally impinge on MN axons or the firm structure of muscle cytoskeleton. Notably, in muscles processed with conventional cryopreservation, residual SNs appear in proximity to blood vessels, which may be explained by uneven freezing-dependent deterioration on SNs in different tissue micro-contexts. While chemical fixation, commonly using aldehyde compounds, overcomes cryopreservation artefacts, it is well known that antigen unmasking in thin sections is a critical step required to exploit the sensitivity of immunostaining for the detection of specific cellular structures or types. Like cryopreservation, antigen unmasking may also lead to tissue deterioration and disappearance of the small and weakly reactive SN structures in the muscle section. In addition, most available markers, used for subtype-specific neuronal identification, are raised against highly diffusible epitopes that require immobilization for native tissue retention. The detection of SNs demands, therefore, to avoid the loss of cellular integrity, as occurring upon cryopreservation or aggressive antigen unmasking processes, on the one hand, and sufficient fixation for the retention of endogenous protein distribution, on the other hand. We previously reported that, in cardiac muscle samples, a brief chemical fixation accompanied by a delicate tissue permeabilization is critical to avoid the loss of immunoreactivity of both neurons and cellular membranes (Prando et al., 2018; Pianca et al., 2019).

By applying such protocols to the study of intact muscle fragments, we showed that SNs innervate the muscle at a density much higher than what would be revealed with conventional tissue processing. Such notable differences between the number of SNs identified in samples processed with these precautions, compared to those processed with standard protocols (cryopreservation), may justify why SN innervation of muscles has only rarely been accounted for. While the study was not designed to address the relationship between sympathetic innervation and the diverse metabolic myofiber subtypes, the comparable density of SN processes, measured in both fast and slow fibers, in different muscles, allowed to exclude that neurons are a primary driver of metabolic fiber type, at least under basal conditions. The highly heterogenous innervation observed between diverse muscle types (Figure 4A) suggests, therefore, to seek for other possible reasons and effects, influencing both structural and functional neuro-muscular interactions. The considerable effect on SN quantification, achieved by finely tuning tissue processing, may also explain how the histopathological inspection of muscle biopsies, using protocols tailored to the analysis of myocyte and MNs, has failed to reveal differences in sympathetic innervation in muscles affected by neuro-muscular diseases, including ALS. In this study, we show that experimental and human muscles harboring the fALS mutation, SOD1^G93A^, feature morphologically altered SNs, with a reduced density of neuronal processes and signs consistent with autonomic neurodegeneration. Although the study is observational, the analysis of murine muscles at different disease stages indicates that SN degeneration is shared among different muscles and sets early during ALS progression, before the appearance of other clinical/pathological signs. Whether SNs degenerate due to a cell autonomous effect of the ALS mutation, or for the secondary effect of the ALS muscle environment on neuronal tropism will specifically be the subsequent research object. However, the evidence that SNs were degenerated in muscles of both ubiquitously restricted and muscle-restricted SOD1^G93A^ transgenic mice, the latter displaying a primary genetic defect selectively in skeletal muscle, supports that mutation-harboring ALS muscles, in addition to MNs (Dobrowolny et al., 2018), may negatively impact sympathetic innervation. Thus, our findings demonstrate that SNs are an additional cell type affected in ALS and directs the efforts toward the understanding of how they may contribute to disease pathogenesis and whether their modulation may positively impact autonomic symptoms, modifying the clinical progression of the disease.

## 5 Limitations

The current study shows that exploitation of refined histopathological assessment identifies sympathetic neuropathology in skeletal muscles of ALS patients and animal models. The study is not designed to reveal the mechanisms of SN degeneration, as it relies on the detection of morpho-structural abnormalities through immunofluorescence, and although supported by accurate tissue morphometry, analyses have been performed on a small number of human biopsies and ALS animal models. As such, results cannot be generalized to all disease genotypes and types, but the concept uncovered here may represent a starting point for deeper research on the mechanisms of neuropathology and more thorough investigation of the cellular basis of autonomic involvement across different ALS forms.

## Data Availability

The raw data supporting the conclusion of this article will be made available by the authors, without undue reservation.

## Abbreviations

ALS, amyotrophic lateral sclerosis; β-ARs, β-adrenergic receptors; f, familial; HRV, heart rate variability; MLC, myosin light chain; MN, motor neuron; NA, noradrenaline; NDP, Neuro Detection Protocol; NMJ, neuro muscular junction; PBS, phosphate-buffered saline; PFA, paraformaldehyde; RT, room temperature; s, sporadic; SAPV, systolic arterial pressure variability; SNs, sympathetic neurons; SOD1, superoxide dismutase 1; TH, tyrosine hydroxylase.
