# Supplementary material for: Sympathetic neuropathology is revealed in muscles affected by amyotrophic lateral sclerosis
Source: Front Physiol. 2023 May 12;14:1165811. doi: 10.3389/fphys.2023.1165811 (PMC10213213; doi:10.3389/fphys.2023.1165811)
Supplement: Supplementary file 1 [file DataSheet1.pdf]

Sympathetic neuropathology is revealed in muscles affected by Amyotrophic Lateral Sclerosis

Antonio Mazzaro [1,2], Veronica Vita [1,2], Marco Ronfini [2,3], Irene Casola [4], Arianna Klein [2], Gabriella Dobrowolny [4], Gianni Sorarù [5], Antonio Musarò [4,6], Marco Mongillo [2,3,7,8,*] & Tania Zaglia [2,3,8,*]

[1], Department of Cardiac, Thoracic, Vascular Sciences and Public Health, University of Padua, via Giustiniani 2, 35131, Padua, Italy.

[2], Veneto Institute of Molecular Medicine, via Orus 2, 35129, Padua, Italy.

[3], Department of Biomedical Sciences, University of Padua, via Ugo Bassi 58/B, 35121, Padua, Italy.

[4], Laboratory Affiliated to Institute Pasteur Italia-Fondazione Cenci Bolognetti, DAHFMO-Unit of Histology and Medical Embryology, Sapienza University of Rome, Via A. Scarpa, 14, 00161 Rome, Italy.

[5], Department of Neuroscience, Azienda Ospedaliera di Padova, Via Giustiniani 2, 35128, Padua, Italy.

[6], Scuola Superiore di Studi Avanzati Sapienza (SSAS), Sapienza University of Rome, 00185 Rome, Italy.

[7], CNR Institute of Neuroscience, Viale G. Colombo 3, 35121 Padua, Italy.

[8], CIR-MYO Myology Center, University of Padova, 35131 Padua, Italy.

***, equal contribution**

Correspondence to:

Tania Zaglia, PhD

Department of Biomedical Sciences, University of Padua, Via Ugo Bassi 58/B, 35121 Padua, Italy

Veneto Institute of Molecular Medicine, via Orus 2, 35129 Padua, Italy

CIR-MYO Myology Center, University of Padova, 35131 Padua, Italy

Tel: +39 0497923294

Fax: +39 0497923250

e-mail: tania.zaglia@unipd.it

Marco Mongillo, MD, PhD

Department of Biomedical Sciences, University of Padua, Via Ugo Bassi 58/B, 35121 Padua, Italy

Veneto Institute of Molecular Medicine, via Orus 2, 35129 Padua, Italy

CIR-MYO Myology Center, University of Padova, 35131 Padua, Italy

Tel: +39 0497923229

Fax: +39 0497923250

e-mail: marco.mongillo@unipd.it

Supplementary Data content:

- Supplementary Figures 1-3;

- Supplementary Figure Legends 1-3;

- Supplementary Table 1;

Figure Legends

Supplementary Figure 1. Histopathology of normal murine muscles processed with Neuron Detection Protocol (NDP).

(**A-B**) Haematoxylin-eosin staining of sections of tibialis anterior, harvested from normal 5-month-old C57BL/6J male mice, either processed with our improved NDP (**A**) or directly frozen in liquid nitrogen (**B**).

Supplementary Figure 2. Detection of sympathetic neuron processes in murine muscles.

Confocal immunofluorescence of tibialis anterior sections, processed with NDP, co-stained with antibodies to tyrosine hydroxylase (TH, red) and neurofilament-H (NF-H, green).

Supplementary Figure 3. Sympathetic innervation topology in murine muscles.

(**A**) Confocal immunofluorescence of tibialis anterior sections, processed with NDP and co-stained with antibodies to tyrosine hydroxylase (TH, purple) and Smooth Muscle Actin (SMA, green), in combination with Cy3-cnjugated phalloidin. (**B**) Confocal immunofluorescence of *Tibialis anterior* sections, processed with NDP, co-stained with antibodies to tyrosine hydroxylase (TH, green) and anti-myosin-IIa (red).

A

NDP, tibialis anterior

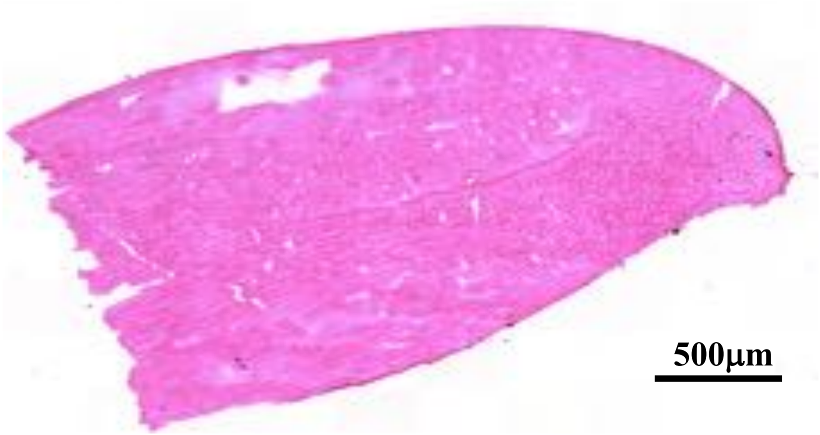

B

cryopreservation, tibialis anterior

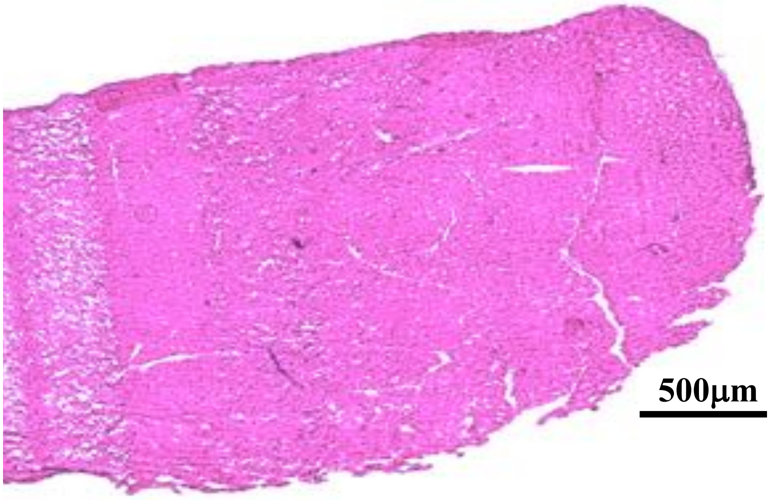

Supplementary Figure 1

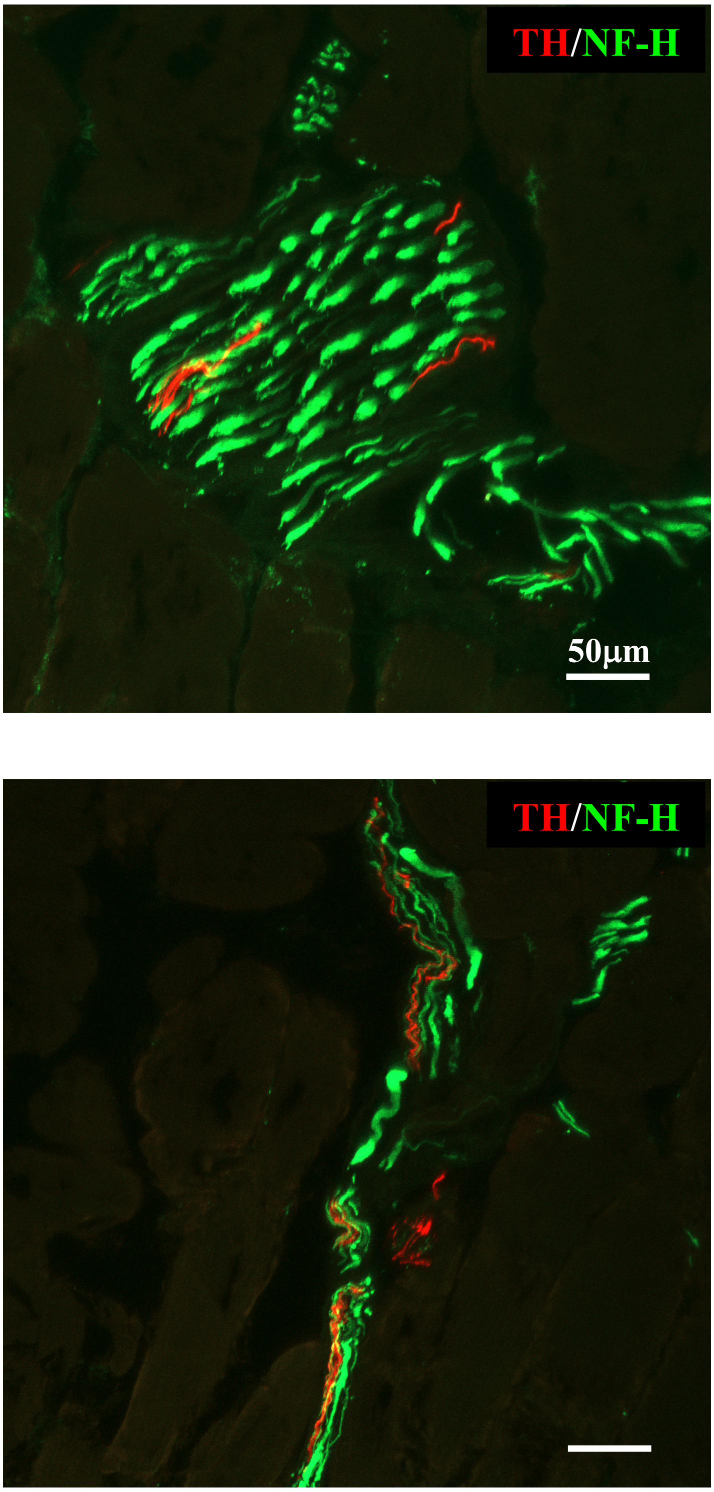

Supplementary Figure 2

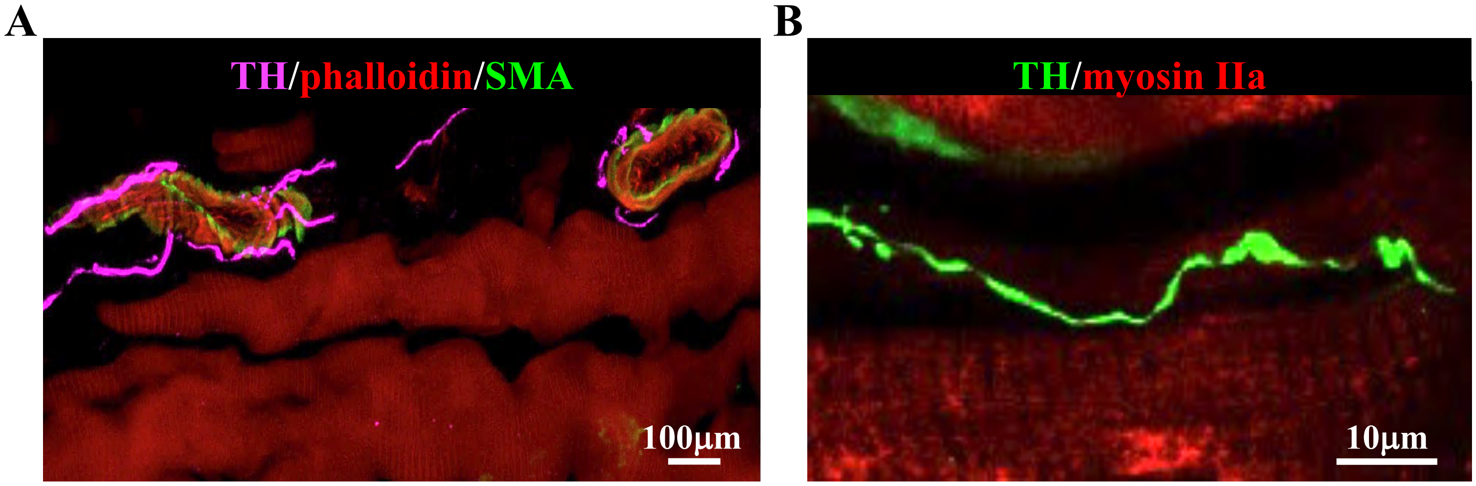

Supplementary Figure 3

Primary Antibodies	Supplier	Code	Diluition
Mouse anti sarcomeric α-actinin	Sigma-Aldrich	A7732	1:200
AlexaFluor 647-conjugated BTX	Invitrogen	B35450	1:1000
Mouse anti Myosin-I	Developmental Studies Hybridoma Bank	BA-D5	1:100
Mouse anti Myosin-IIa	Developmental Studies Hybridoma Bank	SC-71	1:100
Mouse anti Myosin-IIb	Developmental Studies Hybridoma Bank	BF-F3	1:100
Rabbit anti Neurofilament-H	MyBioSource	MBS4152815	1:1000
Alexa Fluor 568-conjugated Phalloidin	Invitrogen	A12380	1:400
Rabbit anti-Tyrosine Hydroxylase	Millipore	Ab152	1:400
FITC-conjugated Smooth Muscle Actin	Invitrogen	A12380	1:400

Supplementary Table 1. List of primary antibodies and dyes used in this study. BTX, bungarotoxin.
